# Lactic Acid Bacteria-Derived Exopolysaccharides Mitigate the Oxidative Response via the NRF2-KEAP1 Pathway in PC12 Cells

**DOI:** 10.3390/cimb45100510

**Published:** 2023-10-02

**Authors:** Seda Şirin

**Affiliations:** Department of Biology, Faculty of Science, Gazi University, Teknikokullar, 06500 Ankara, Turkey; sdasirin@hotmail.com

**Keywords:** exopolysaccharide, hydrogen peroxide, lactic acid bacteria, oxidative stress, NRF2-KEAP1 pathway, PC12 cell

## Abstract

Parabiotics, including L-EPSs, have been administered to patients with neurodegenerative disorders. However, the antioxidant properties of L-EPSs against H_2_O_2_-induced oxidative stress in PC12 cells have not been studied. Herein, we aimed to investigate the antioxidant properties of the L-EPSs, their plausible targets, and their mechanism of action. We first determined the amount of L-EPSs in *Lactobacillus delbrueckii* ssp. *bulgaricus* B3 and *Lactiplantibacillus plantarum* GD2 using spectrophotometry. Afterwards, we studied their effects on TDH, TOS/TAS, antioxidant enzyme activities, and intracellular ROS level. Finally, we used qRT-PCR and ELISA to determine the effects of L-EPSs on the NRF2-KEAP1 pathway. According to our results, the L-EPS groups exhibited significantly higher total thiol activity, native thiol activity, disulfide activity, TAS levels, antioxidant enzyme levels, and gene expression levels (GCLC, HO-1, NRF2, and NQO1) than did the H_2_O_2_ group. Additionally, the L-EPS groups caused significant reductions in TOS levels and KEAP1 gene expression levels compared with those in the H_2_O_2_ group. Our results indicate that H_2_O_2_-induced oxidative stress was modified by L-EPSs. Thus, we revealed that L-EPSs, which regulate H_2_O_2_-induced oxidative stress, could have applications in the field of neurochemistry.

## 1. Introduction

Neurodegenerative diseases (NDs), such as Alzheimer’s disease (AD), Parkinson’s disease (PD), Huntington’s disease (HD), amyotrophic lateral sclerosis (ALS), and multiple sclerosis (MS), form a non-homogenous neurological disease group characterized by neuronal loss in the central nervous system (CNS) or peripheral nervous system (PNS), with negative impacts of the lives of millions of people across the globe [1,2]. NDs both emerge and progress through body events in which oxidative stress (OS) plays a pivotal role [3]. OS is characterized by a state in which total oxidant status (TOS) surpasses total antioxidant status (TAS), leading to TOS/TAS imbalance and injury through reactive oxygen species (ROS) [4]. Dynamic thiol-disulfide homeostasis (TDH) is defined as the ratio of the thiol parameters to the disulfide parameters; it also provides an insight into the TOS/TAS imbalance [5]. Nuclear factor erythroid 2-related factor 2 (NRF2) is believed to be the key regulator of TOS/TAS balance inside cells. Under non-pathological conditions, cytoplasmic NRF2 expression is carried out by its endogenous inhibitor Kelch-like ECH-associated protein 1 (KEAP1). In the case of cellular OS, NRF2 can escape degradation by KEAP1, passing into the nucleus. There, it leads to increased transcription of the antioxidant genes, which include catalase (CAT), glutamate cysteine ligase catalytic (GCLC), glutathione peroxidase (GPx), heme oxygenase-1 (HO-1), NAD(P)H quinone dehydrogenase 1 (NQO1), paraoxonase-1 (PON-1), and superoxide dismutase (SOD); it fulfills this task by binding to the antioxidant response element (ARE) found in their promoter region [6,7]. Unfortunately, the available treatments neither prevent the occurrence of NDs, nor slow down or cure them, although they provide some symptomatic relief. Although it has been asserted that OS plays a role in the development of NDs, no modern widely available treatment specifically focuses on OS. Natural antioxidants involving CNS or PNS have recently drawn considerable attention in the treatment of OS [8]. One such example is postbiotics [9]. These constitute a novel introduction into the field after probiotics and prebiotics, and primarily denote host-friendly metabolites of the microbes, bacterial composition, and the inactivated bacteria themselves. Compared with living bacteria, postbiotics offer a better stability, absorption mechanics, and metabolic processing, which collectively ensure their enduring efficacy while being stored and used. Exopolysaccharides (EPSs), cell-free fermentation filtrate, bacteriostats, antioxidant peptides, antibacterial peptides, antioxidant enzymes, and other active ingredients represent the most commonly utilized postbiotic categories. EPSs are also exhibit substantial antioxidant, antimicrobial, and immunomodulatory activities, enabling them to modulate certain physiological reactions by interacting with various molecules or receptors or exploiting various mechanisms employing microbiota homeostasis or host metabolic and signaling pathways [10]. 

Various studies in the literature have demonstrated that EPSs exert regulatory effects on the NRF2-KEAP1 signaling pathway. Wang et al. [11] demonstrated that EPSs (EPS103) originated from *Lactiplantibacillus plantarum* JLAU103 showed protective effects in immune-suppressed mice against OS induced by cyclophosphamide through activation of antioxidation enzymes and the NRF2/KEAP1 pathway. Zhang et al. [10] showed that EPSs contained in the *Bacillus amyloliquefaciens* lysate (BAL1) increased the nuclear displacement of NRF2 and thus triggered the transcription of downstream antioxidant enzyme genes in human embryonic fibroblasts (CCC-ESF-1); in this way, they boosted the antioxidant capacity of antioxidant enzymes to remove the excess amounts of intracellular ROS. 

To date, no study has yet demonstrated that EPSs show regulatory effects on the NRF2-KEAP1 signaling pathway in the neurodegenerative cell model (H_2_O_2_-induced OS). Herein, we aimed to show the regulatory effects of EPSs produced by the probiotic strains *Lactobacillus delbrueckii* ssp. *bulgaricus* B3 and *Lactiplantibacillus plantarum* GD2 on H_2_O_2_-induced OS, executed through the NRF2/KEAP1 signaling pathway.

## 2. Materials and Methods

### 2.1. Bacterial Strains Media and Culture Conditions

In this research, *Lactobacillus* strains previously identified from yogurt (*Lactobacillus delbrueckii* ssp. *bulgaricus* B3) and infant feces (*Lactiplantibacillus plantarum* GD2) were utilized. The Lactobacilli were stored at −30 °C in MRS broth (Oxoid, Basingstoke, UK) supplemented with 10% (*v*/*v*) glycerol (Sigma-Aldrich, St. Louis, MO, USA). For experimental use, cultures were revived from these frozen stocks by undergoing two consecutive transfers in MRS broth, followed by incubation at 37 °C for 18 h in an aerobic environment [12].

### 2.2. Isolation of EPSs

The cultures were subjected to boiling at 100 °C for 15 min. Following cooling of the cultures, the cell suspension was subjected to treatment with 17% (*v*/*v*) of 85% trichloroacetic acid (TCA) (Merck Millipore, Burlington, MA, USA) solution and centrifugation (Nuve, Ankara, Turkey) at 21,500× *g* for 20 min to eliminate cells and proteins. EPSs were precipitated using one volume of cold absolute ethanol (Sigma-Aldrich) and centrifuged at 21,500× *g* for a duration of 15 min. The initial step involved fractionating a 20 mg/mL solution of crude EPS (5 mL) through an anion exchange chromatography process using a DEAE-cellulose column measuring 26 cm × 40 cm. Elution was initially carried out with deionized water and subsequently with 0.2 mol/L and 0.5 mol/L NaCl solutions at a flow rate of 1 mL/min. The automated collection of 5 mL fractions was achieved, and the carbohydrate content was quantified using the phenol-sulfuric acid method (Merck-Millipore). Fractions containing polysaccharides were combined, subjected to dialysis, and then freeze-dried for further refinement through gel permeation chromatography on a Sepharose CL-6B column measuring 25 cm × 50 cm. Elution was performed using 0.9% (*w*/*v*) NaCl at a flow rate of 0.5 mL/min. The fractions containing the EPS were pooled, dialyzed, and lyophilized to obtain the purified lyophilized EPSs (L-EPSs) sample. To verify the purity of this sample, UV-vis spectroscopy was employed with a UV-vis spectrophotometer. For UV-vis measurement, the L-EPSs were created by suspending the sample in distilled water, and the wavelength range assessed was 190–550 nm [13,14,15].

### 2.3. The Cell Line Used in the Study

PC-12 is a cell line found in rats which is derived from transplantable pheochromocytoma. The PC-12 cell line was obtained from Bilkent University National Nanotechnology Research Center (UNAM).

### 2.4. Reproduction and Storage of PC-12 Cells

DMEM (Sigma-Aldrich), 10% heat-inactivated horse serum (Thermo Fischer Scientific, Waltham, MA, USA), 10% heat-inactivated fetal bovine serum (Sigma-Aldrich), 1% penicillin/streptomycin (Sigma-Aldrich), and 1% L-glutamine (Sigma-Aldrich) were used to develop the PC-12 cells. They were sterilized by passing them through a 0.22 µm Millipore filter. A total of 10 g/mL collagen (Thermo Fischer) was obtained by dissolving the solution in PBS and applying to the 25 and 75 cm^2^ culture plates (Corning Star, Corning, NY, USA) used for cell growth. After incubation for 10 min, collagen-coated flasks were planted with cells at a density of 2 × 10^5^ or 6 × 10^5^ cells/cm^2^. An incubator (Sanyo, Osaka, Japan) with 5% carbon dioxide (CO_2_) content was used to grow cells at 37 °C. The cell growth medium was lifted out of the flasks after they reached an 80–90% density to count the number of cells within. As per study protocol, by transferring them to 96- and 6-well microplates (Corning Star), a certain number of cells were created. Cells contained in the storage medium, which were prepared with PC-12 cell culture medium and 10% dimethyl sulfoxide (DMSO) (Millipore, Darmstadt, Germany), were stored in a liquid nitrogen tank (Cryogenics, Shinagawa, Tokyo) [16,17,18].

### 2.5. Cell Viability Test

The cell viability of H_2_O_2_ on PC-12 cells was tested in this experiment by applying 200 M H_2_O_2_ to cells at a density of 10^4^ cells/well in a 96-well plate for 24 h. L-EPSs at concentrations of 100, 250, 500, 1000, and 1250 µg/mL found in a solution of 100 µL of cell culture medium with a DMSO content of 0.05% were applied for a period of 24 h. The impact of L-EPSs and H_2_O_2_ treatments on PC-12 cell survival was also investigated, as L-EPSs and H_2_O_2_ will be applied to PC-12 cells in our experimental model. The PC-12 cells were subjected to a 24 h treatment with the prespecified L-EPSs concentrations (100, 250, 500, 1000, and 1250 µg/mL), which was followed by the application of 200 μM H_2_O_2_ for 24 h. 

The 3-(4,5-dimethyl-2-thiazol-2-yl)-2,5-diphenyl-2H tetrazoliumbromide (MTT) test was used to compare the cell viability with that of the control. After the completion of incubation, 20 µL MTT (Sigma-Aldrich) (5 mg/mL dH_2_O) was added into each well. The 96-well plate was incubated for 4 h at 37 °C with 5% CO_2_ before the liquid in the wells was removed, and 200 µL DMSO was added to each well. After the plate was transferred into an incubator with 5% CO_2_ content at 37 °C for 30 min, a microplate reader was used to read the optical density at 570 nm. The absorbance value of the cell culture control group was considered 100% viable; then, using that value, the formula below was used to calculate the percent viability of other cell groups: cell viability (%): mean sample absorbance/mean control absorbance × 100 [19,20].

### 2.6. The Effect of L-EPSs on TDH in PC-12 Cells Induced by OS with H_2_O_2_

A native thiol assay (Rel Assay, Gaziantep, Turkey) and total thiol assay (Rel Assay) were employed to determine TDH, as per the manufacturer’s instructions. For the TDH, free functional thiol groups were created by reducing the reducible disulfide bonds. Unused reductant sodium borohydride was consumed and discarded with formaldehyde; the amount of all thiol groups, both reduced and native, was determined after treatment with 5,5′-dithiobis-(2-nitrobenzoic) acid (DTNB). The dynamic disulfide amount was calculated using the half of the difference between the total thiols and native thiols. The amount of disulfide was ascertained following the determination of the native thiols and total thiols [21].

### 2.7. Determination of the Effect of L-EPSs on Total Oxidant Status (TOS), and Total Antioxidant Status (TAS) Levels in PC-12 Cells Induced by OS with H_2_O_2_

A density of 1 × 10^6^ cells per well was used to seed a 6-well plate. For 24 h at 37 °C and 5% CO_2_, L-EPSs were incubated with PC-12 cells in 2 mL of cell culture media at concentrations of 100, 250, 500, 1000, and 1250 g/mL. Thereafter, 24 h cell incubation was carried out in a cell culture medium containing 200 µM H_2_O_2_. The control group was composed of cells cultured in standard cell culture conditions, without being exposed to H_2_O_2_ or L-EPSs. The cells were washed with cold PBS following the above treatments. Afterwards, the cells were subject to treatment with cold PBS at a volume of 2 mL and scraped with a cell scraper (Corning Star). They were then placed into sterile tubes, which were centrifuged at 10,000 rpm for 5 min at 4 °C. All of the following stages of the applications were performed on ice. A total of 500 liters of cold thawing buffer (1 M Tris buffer (10 mL) pH 7.2 (Merck), 2 M sodium chloride (NaCl), 1 M magnesium chloride (MgCl_2_), and 1% Triton X-100 (Merck)) were applied to the cell pellets. By vortexing the tubes every five minutes, they were kept at 4 °C for 15 min. After this step, the tubes were centrifuged at 10,000× *g* for 30 min at 4 °C to remove the cell debris. Then, the supernatant was collected, and as per the manufacturer’s instructions, the TOS and TAS levels were quantified using the TOS (Rel Assay) and TAS (Rel Assay) kits. The color intensity corresponding to the amount of oxidants in each experimental group was measured by spectrophotometric analysis of the TOS level at 530 nm. The experiment used H_2_O_2_ as its calibration gas, and the results were expressed as moles of H_2_O_2_ equivalent per liter [22,23]. The reduction capacity of the antioxidants in each experimental group for 2,2′-azino-bis(3-ethylbenzothiazoline-6-sulfonic acid (ABTS) measured at 660 nm was used to measure the TAS level. The results are given in units of mmol Trolox equivalent/L [23,24,25].

### 2.8. Determination of the Effect of L-EPSs on Paraoxanase-1 (PON1), Superoxide Dismutase (SOD), Catalase (CAT), and Glutathione Peroxidase (GPx) Antioxidant Enzyme Levels in PC-12 Cells Induced by OS with H_2_O_2_

As per the manufacturer’s instructions, PON1 (Rel Assay), SOD (FineTest, Boulder, CO, USA), CAT (FineTest), and GPX (FineTest) kits were used to quantify PON1, SOD, CAT, and GPx enzyme levels. The paraoxon hydrolysis rate was determined by increased absorbance at 412 nm at 25 °C, brought about by the formation of p-nitrophenol. PON1 activity was described as the production of 1 mmol p-nitrophenol each minute and expressed as U/L [26]. Optical density (OD) was determined at 450 nm on a microplate reader to quantify SOD, CAT, and GPx enzyme activity. In each experimental group, the numbers corresponding to the measurements were reported as percent SOD, CAT, and GPX enzyme levels, as compared with the control [27].

### 2.9. Determination of the Effect of L-EPSs on Intracellular ROS Level in PC-12 Cells Induced by OS with H_2_O_2_ by Flow Cytometry

After the cells were treated with L-EPSs plus H_2_O_2_, they were further treated with DCFDA (20 μM) (Cayman Chemical, Ann Arbor, MI, USA), as described in the preceding section. The cells were first cultured at 37 °C for 30 min, pipetted, and submitted to flow cytometry (ACEA NovoCyte, Santa Clara, CA, USA). The number of analyzed cells in each sample was 10.000; we then analyzed the change in ROS using the digital program (NovoExpress, Santa Clara, CA, USA).

### 2.10. Determination of the Effect of L-EPSs on GCLC, HO-1, NRF2, NQO1, and KEAP1 Gene Expression Levels in PC-12 Cells Induced by OS with H_2_O_2_ by qRT-PCR

After the cells were treated as per the manufacturer’s instructions, the total cellular RNA extraction was carried out with the RNeasy Mini kit (Qiagen, Hilden, Germany). The next step was the conversion of RNA to DNA, with the help of the QuantiTect Reverse Transcription Kit from Qiagen. cDNA synthesis and PCR reaction from extracted RNA samples were carried out with a QuantStudio 3 Real-Time PCR instrument from Applied Biosystems (Thermo-Fischer Scientific). The primer sequences are presented on Table 1. The settings below were employed for the application of qRT-PCR: 5 min at 95 °C for 1 cycle; 40 cycles of 95 °C for 10 s; 56 °C (NQO1) and 60 °C (GCLC, HO-1, KEAP, and NRF2) for 30 s; and 72 °C for 45 s. In order to complete the final extension step, one cycle at 72 °C for 30 s was performed. The relative level of expression for mRNA was gauged using glyceraldehyde-3-phosphate dehydrogenase (GAPDH).

### 2.11. Determination of GCLC, HO-1, KEAP, NRF2, and NQO1 by an ELISA Assay

The levels of cell lysates of GCLC, HO-1, KEAP, NRF2, and NQO1 were computed using an ELISA assay kit (Abcam, Cambridge, UK). All steps were carried out as per the manufacturer’s instructions.

### 2.12. Statistics

Various treatments were compared using a one-way analysis of variance (ANOVA) test with a post hoc Tukey’s HSD test. Study results were reported as the mean ± standard error (SD) of the mean for each group and were considered statistically significant at a confidence level of 95% and a statistical significance of *p* < 0.05. All statistical analyses were performed using SPSS (Version 21, IBM, Armonk, NY, USA).

## 3. Results

### 3.1. Purity and Amount of L-EPSs

The UV-vis spectroscopy results of the L-EPSs revealed no absorption peaks at 260 or 280 nm, showing the absence of proteins and nucleic acids in the purified L-EPSs. The amounts of L-EPSs produced by *L. delbrueckii* ssp. bulgaricus B3 and *L. plantarum* GD2 were 435 and 385 mg/L, respectively.

### 3.2. Protective Effect of L-EPSs in PC-12 Cells Induced by OS with H_2_O_2_

Whereas 200 μM H_2_O_2_ applied for 24 h significantly reduced cell viability to 50% (*p* < 0.05), 100, 250, 500, 1000, and 1250 μg/mL L-EPSs applied for 24 h increased the cell survival rate to 60–90% and significantly inhibited the cellular damage mediated by H_2_O_2_ (*p* < 0.05). *L. delbrueckii* ssp. bulgaricus B3 and *L. plantarum* GD2 L-EPSs provided cell viability rates of 66%, 72%, 76%, 84%, 90% and 60%, 67%, 71%, 75%, and 81% at concentrations of 100, 250, 500, 1000, and 1250 μg/mL, respectively. The L-EPSs showed no cytotoxic effects at the above concentrations. 

### 3.3. Regulatory Effect of L-EPSs on TDH in PC-12 Cells Induced by OS with H_2_O_2_

**Figure 1:**
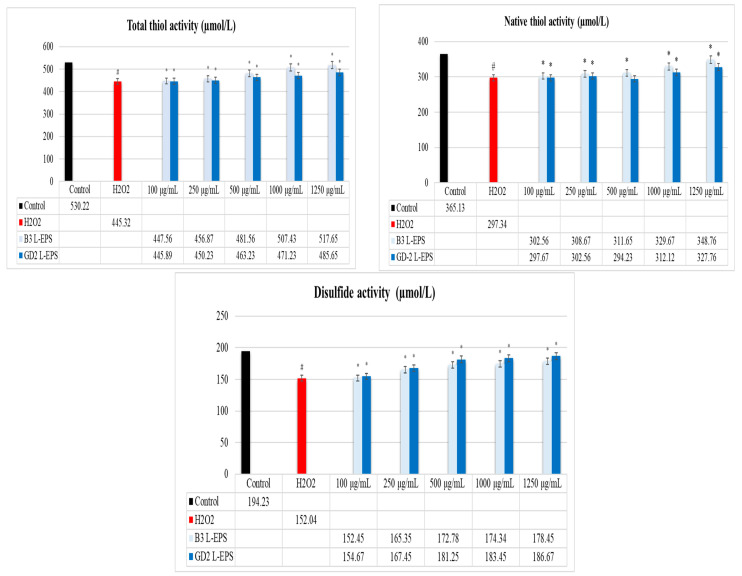
Levels of total thiol, native thiol, and disulfide. The figures were presented as mean ± SD. Tukey’s test was used in the case of *p* < 0.05. ^#^ Significantly different from the control (*n*:3 for each bar). * Significantly different from the H_2_O_2_ (*n*:3 for each bar).

The results of this experiment are given in Figure 1. The total thiol activity, native thiol activity, and disulfide activity were significantly lower in the H_2_O_2_ group (445.32 µmol/L; 297.34 µmol/L; 152.04 µmol/L, respectively) compared with the controls (530.22 µmol/L; 365.13 µmol/L; 194.23 µmol/L, respectively) (*p* < 0.05). In a concentration-dependent manner, the L-EPS groups (445.89–517.65 µmol/L; 297.67–348.76 µmol/L; 152.45–186.67 µmol/L, respectively) showed significantly higher total thiol activity, native thiol activity, and disulfide activity than did the H_2_O_2_ group (445.32 µmol/L; 297.34 µmol/L; 152.04 µmol/L, respectively) (*p* < 0.05). The efficacies of the L-EPS concentrations in descending order were 1250 µg/mL > 1000 µg/mL > 500 µg/mL > 250 µg/mL > 100 µg/mL. The strain-based order of action of the L-EPSs on disulfide activity was GD2 > B3; on the other hand, the strain-based order of action of the L-EPSs on total thiol activity and native thiol activity was B3 > GD2. 

### 3.4. Regulatory Effect of L-EPSs on TOS and TAS Levels in PC-12 Cells Induced by OS with H_2_O_2_

**Figure 2:**
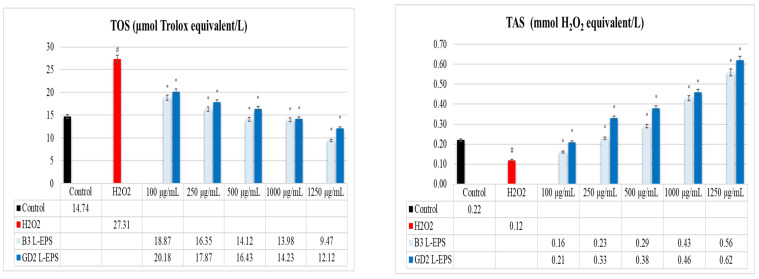
Levels of TOS and TAS. The figures were presented as mean ± SD. Tukey’s test was used in the case of *p* < 0.05. ^#^ Significantly different from the control (*n*:3 for each bar). * Significantly different from the H_2_O_2_ (*n*:3 for each bar).

The results of this experiment are given in Figure 2. The H_2_O_2_ group (27.31 µmol Trolox equivalent/L; 0.12 mmol H_2_O_2_ equivalent/L, respectively) showed a significantly higher TOS level and a significantly lower TAS level than those found in the controls (14.74 µmol Trolox equivalent/L; 0.22 mmol H_2_O_2_ equivalent/L, respectively). However, the L-EPSs groups (9.47–20.18 µmol Trolox equivalent/L) led to a significant reduction in TOS level in the H_2_O_2_ group (27.31 µmol Trolox equivalent/L) in a concentration-based manner (*p* < 0.05). Based on their concentration, the L-EPS groups (0.16–0.62 mmol H_2_O_2_ equivalent/L) caused a significant increment in TAS levels compared with the H_2_O_2_ group (0.12 mmol H_2_O_2_ equivalent/L) (*p* < 0.05). The efficacies of the L-EPS concentrations in descending order were 1250 µg/mL > 1000 µg/mL > 500 µg/mL > 250 µg/mL > 100 µg/mL. The strain-based efficacy of the L-EPSs was B3 > GD2 for TOS and GD2 > B3 for TAS. 

### 3.5. Inductive Effect of L-EPSs on PON1, SOD, CAT, and GPx Antioxidant Enzyme Levels in PC-12 Cells Induced by OS with H_2_O_2_

**Figure 3:**
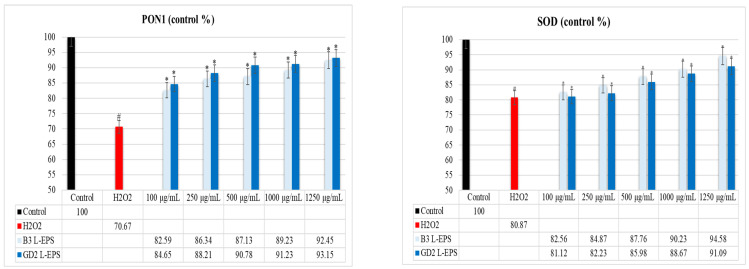

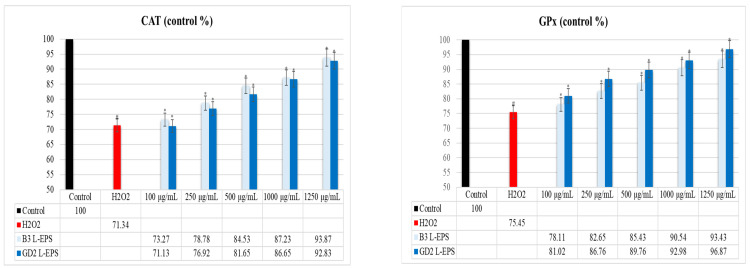
Levels of the antioxidant enzymes PON1, SOD, CAT, and GPx. The figures were presented as mean ± SD. Tukey’s test was used in the case of *p* < 0.05. ^#^ Significantly different from the control (*n*:3 for each bar). * Significantly different from the H_2_O_2_ (*n*:3 for each bar).

The results of the experiment are given in Figure 3. PON1, SOD, CAT, and GPx antioxidant enzyme levels were found to be significantly lower in the H_2_O_2_ group (70.67%; 80.87%; 71.34%; 75.45%, respectively) compared with the control group (100%; 100%; 100%; 100%, respectively) (*p* < 0.05). On the contrary, the PON1, SOD, CAT, and GPx antioxidant enzyme levels were considerably increased by the L-EPS groups (82.59–93.15%; 81.12–94.58%; 71.13–93.87%; 78.11–96.87%, respectively) in a concentration-based manner as compared with the levels in the H_2_O_2_ group (70.67%; 80.87%; 71.34%; 75.45%, respectively) (*p* < 0.05). The efficacy of the L-EPSs concentrations in descending order was 1250 µg/mL > 1000 µg/mL > 500 µg/mL > 250 g/mL > 100 g/mL. The efficacy of the L-EPSs by strain was as follows: B3 was prominent in SOD and CAT, while GD2 was prominent in PON1 and GPx. The antioxidant enzyme levels affected by the L-EPS groups was, in descending order, SOD > CAT > GPx > PON1. 

### 3.6. Inhibitory Effect of L-EPSs on Intracellular ROS Level in PC-12 Cells Induced by OS with H_2_O_2_

**Figure 4:**
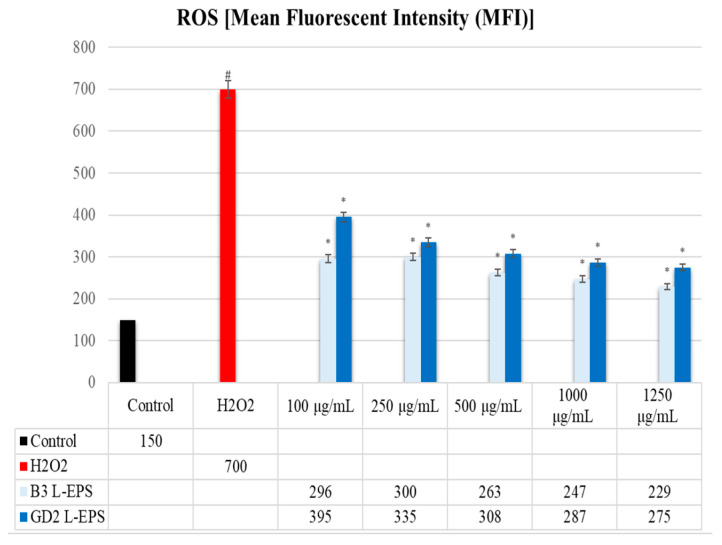
Levels of intracellular ROS. The figures were presented as mean ± SD. Tukey’s test was used in the case of *p* < 0.05. ^#^ Significantly different from the control (*n*:3 for each bar). * Significantly different from the H_2_O_2_ (*n*:3 for each bar).

The results are of the experiment are given in Figure 4. The intracellular ROS level was found to be significantly higher in the H_2_O_2_ group (700 MFI) compared with the control group (150 MFI) (*p* < 0.05). In contrast, the intracellular ROS level was considerably decreased by the L-EPS groups (229–395 MFI) in a concentration-based manner as compared with the levels in the H_2_O_2_ group (700 MFI) (*p* < 0.05). The efficacy of the L-EPSs concentrations, in descending order, were 1250 µg/mL > 1000 µg/mL > 500 µg/mL > 250 g/mL > 100 g/mL. The efficacy of the L-EPSs by strain was as follows: B3 > GD2.

### 3.7. Regulatory Effect of L-EPSs on GCLC, HO-1, NRF2, NQO1, and KEAP1 Gene Expression Levels in PC-12 Cells Induced by OS with H_2_O_2_

**Figure 5:**
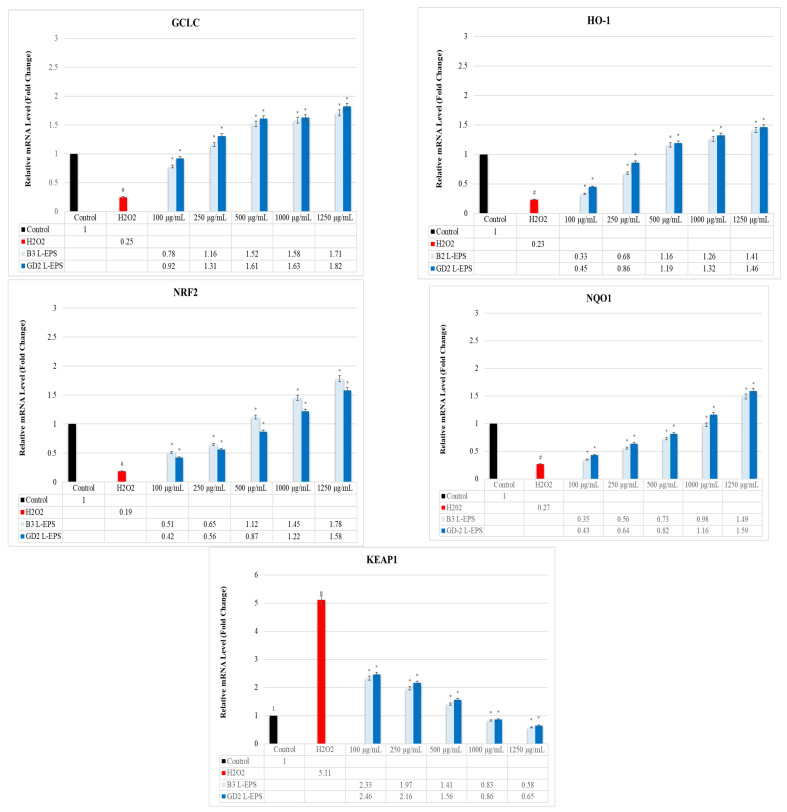
The expression levels of the genes GCLC, HO-1, NRF2, NQO1, and KEAP1. The figures were presented as mean ± SD. Tukey’s test was used in the case of *p* < 0.05. ^#^ Significantly different from the control (*n*:3 for each bar). * Significantly different from the H_2_O_2_ (*n*:3 for each bar).

The results of the experiment are given in Figure 5. The H_2_O_2_ group (0.25 fold; 0.23 fold; 0.19 fold; 0.27 fold, respectively) exhibited significantly lower GCLC, HO-1, NRF2, and NQO1 gene expression levels than did the controls (1 fold; 1 fold; 1 fold; 1 fold, respectively) (*p* < 0.05). This reduction was significantly increased by the L-EPS groups (0.78–1.82 fold; 0.33–1.46 fold; 0.42–1.78 fold; 0.35–1.59 fold, respectively), based on their concentrations (*p* < 0.05). The H_2_O_2_ group (5.11 fold) showed a significantly increased KEAP1 gene expression level than did the control (1 fold) (*p* < 0.05). This increase was significantly decreased by the L-EPSs groups (0.58–2.46 fold), based on their concentration (*p* < 0.05). The effectiveness of the L-EPSs concentrations were in the descending order of 1250 µg/mL > 1000 µg/mL > 500 µg/mL > 250 µg/mL > 100 µg/mL; on the other hand, the effectiveness of the L-EPSs by strain was as follows: B3 L-EPS was prominent in NRF2 and KEAP1, while GD2 L-EPS was prominent in GCLC, HO-1, and NQO1. The gene expression levels affected by the L-EPS groups were, in descending order, NRF2 > KEAP1 > GCLC > HO-1 > NQO1.

### 3.8. Regulatory Effect of L-EPSs on GCLC, HO-1, NRF2, NQO1, and KEAP1 Protein Expression Levels in PC-12 Cells Induced by OS with H_2_O_2_

The results of the experiment are given in Table 2. The H_2_O_2_ group (30%; 33%; 25%; 36%, respectively) exhibited significantly lower GCLC, HO-1, NRF2, and NQO1 protein expression levels than did the controls (100%; 100%; 100%; 100%, respectively) (*p* < 0.05). This reduction was significantly increased by the L-EPS groups (33–98%; 34–93%; 34–106%; 36–89%, respectively), based on their concentrations (*p* < 0.05). The H_2_O_2_ group (225%) showed a significantly increased KEAP1 protein expression level than did the control (100%) (*p* < 0.05). This increase was significantly decreased by the L-EPSs groups (98–220%), based on their concentration (*p* < 0.05). The effectiveness of the L-EPSs concentrations were in the descending order of 1250 µg/mL > 1000 µg/mL > 500 µg/mL > 250 µg/mL > 100 µg/mL; on the other hand, the effectiveness of the L-EPSs by strain was as follows: B3 L-EPS was prominent in NRF2, and KEAP1, while GD2 L-EPS was prominent in GCLC, HO-1, and NQO1. The protein expression levels affected by the L-EPS groups were in descending order of NRF2 > KEAP1 > GCLC > HO-1 > NQO1. The results we obtained from qRT-PCR are similar to the results we obtained from ELISA.

## 4. Discussion

The role of OS in the onset and progression of numerous NDs like AD, PD, HD, ALS, and MS has been widely acknowledged [28]. Current therapeutic approaches for these NDs are primarily palliative in nature, focusing on symptom management rather than on treating the root cause. Commonly prescribed drugs for these diseases include donepezil, rivastigmine, galantamine, and tacrine [29]. However, their inability to effectively halt or reverse the progression of these diseases has necessitated the exploration of alternative treatments. In this context, the spotlight has turned to postbiotics, which are essentially beneficial products derived from probiotic bacteria following their metabolic activities [9]. Notably, EPSs derived from lactic acid bacteria, termed L-EPSs, have emerged as promising candidates due to their potent antioxidant properties [10]. These L-EPSs may counteract the detrimental effects of OS and potentially slow down or reverse the damage resulting from neurodegenerative conditions. In the present study, we delved deeper into the therapeutic potential of L-EPSs. We specifically studied their ability to mitigate H_2_O_2_-induced OS in PC12 cells, which serve as a well-established model for neuronal oxidative damage. Our investigation centered around the NRF2-KEAP1 signaling pathway, a critical regulator of cellular antioxidant response. By understanding the interactions and modulatory effects of L-EPSs on this pathway, we hoped to shed light on their mechanism of action and pave the way for novel, effective treatments for NDs.

The structural attributes of L-EPSs are believed to contribute to their antioxidant activity. Previously, our team analyzed the monosaccharide composition of EPSs from *L. delbrueckii* ssp. *bulgaricus* B3 and *L. plantarum* GD2 using HPLC. We found that *L. delbrueckii* ssp. *bulgaricus* B3 L-EPS contained mannose (88.25%), glucose (9.54%), a combination of sucrose and maltose (1.10%), fructose (1.04%), and n-acetyl glucosamine (0.07%). Meanwhile, *L. plantarum* GD2 L-EPS contained mannose (71.03%), glucose (25.97%), arabinose (2.73%), and n-acetyl glucosamine (0.27%) [30]. Mannose was the major component in L-EPSs. Gientka et al. [31] observed that EPSs from yeast species, with mannose content exceeding 50%, exhibited pronounced antioxidant activities.

Based on our past studies, the molecular weights of L-EPSs from *L. delbrueckii* ssp. *bulgaricus* B3 was 1.2 × 10^4^ and 3.5 × 10^2^ Da, and for *L. plantarum* GD2 was 2.4 × 10^3^ and 2.3 × 10^2^ Da [30]. Li et al. [32] suggested that EPSs with lower molecular weights (≤10^4^ Da) penetrate host cell membranes with ease. In contrast, Cheng et al. [33] noted that EPSs with higher molecular weights exhibit potent antioxidant, antitumor, immunomodulatory, and neuroprotective activities. Supporting this, Ayyash et al. [34] found an EPS from a novel *L. plantarum* C70, isolated from camel milk, with a high molecular weight of 3.8 × 10^5^ Da, which exhibited robust bioactivity. Further evidence from Cheng et al. [33] hinted at the potential neuroprotective properties of high molecular weight polysaccharides from *Hericium erinaceus* (HE).

The FTIR analysis of L-EPSs indicated the presence of numerous O-H groups within the polysaccharide ring. Broad stretches near 3300 cm^−1^ pointed to hydroxyl group vibrations in carbohydrates. Typical hexoses, like glucose and galactose, as well as deoxyhexoses, such as rhamnose, displayed C-H stretching, seen around the 2850–2960 cm^−1^ band. The regions around 1735 cm^−1^ and 1650–1660 cm^−1^ were attributed to C=O vibrations, and the range from 800 to 1200 cm^−1^, known as the fingerprint region, distinguishes various polysaccharides. Our prior work highlighted a specific band around 836 cm^−1^ associated with α-D glucan [35]. These hydroxyl, amino, carbonyl, and carboxyl groups (polyanionic functional groups) are known to influence the functionalities and antioxidant capabilities of EPSs [36,37].

Previously, we highlighted the rough and porous surface of L-EPSs from *L. delbrueckii* ssp. *bulgaricus* B3 and *L. plantarum* GD2 [35]. Such surfaces enhance the EPSs ability to bind to diverse surfaces, possibly increasing their antioxidant potential. It has been noted that a coarse surface enables more efficient cellular binding.

Our past findings also suggest that the L-EPSs from these strains display a predominantly amorphous or weakly crystalline nature [35]. Generally, amorphous substances dissolve faster, enhancing bioavailability, and are believed to exhibit superior biological activity to that of their crystalline counterparts. This suggests that EPSs with amorphous properties might have better biological functionality and, consequently, stronger antioxidant activities.

In conclusion, factors like a high mannose content, high molecular weight, the presence of polyanionic functional groups, a rough surface, and the amorphous structure of L-EPSs are all associated with their antioxidant abilities. Our team has previously confirmed the strong antioxidant potential of these L-EPSs, as shown by various activity metrics [35].

TDH is of utmost importance, due to its ability to evaluate the redox disequilibrium [38]. Thiols constitute an essential part of antioxidant buffer systems. They possess the SH groups that enable them to prevent OS by reacting and depleting oxidant molecules. As a result of this chemical reaction, thiols are transformed into disulfide in a reversible manner to maintain the dynamic TDH pattern [39]. In NDs, an imbalance affecting ROS alters the function of cysteines and disrupts the integrity of TDH [40]. We thus used commercial kits to investigate how L-EPSs would affect TDH in PC-12 cells with H_2_O_2_-induced OS (in vitro ND model). In our study, the total thiol activity, native thiol activity, and disulfide activity were significantly lower in the H_2_O_2_ group compared with those observed in the controls (*p* < 0.05). The L-EPS groups showed significantly higher total thiol activity, native thiol activity, and disulfide activity than did the H_2_O_2_ group (*p* < 0.05). As Gumusyayla et al. [41] and Vural et al. [39] reported, patients with NDs show significantly lower total thiol activities and native thiol activities than does the healthy population. The low total thiol activities and natural thiol activities observed in the H_2_O_2_ group in our study are similar to those reported by Gümüşyayla et al. [41] and Vural et al. [39] in patients with NDs. However, while the study of Gümüşyayla et al. [41] and Vural et al. [39] included patients with NDs, our study included PC-12 cells induced by OS with H_2_O_2_ (in vitro ND model). Our study showed, for the first time, how L-EPSs acted on TDH in PC-12 cells induced by OS with H_2_O_2_. It was thus not possible to make a comparison with the studies in the literature. The studies in the literature focus on the effects of probiotics on TDH, but these studies are not related to NDs [42,43]. Based on our findings, there is a regulatory effect of L-EPSs on TDH in PC-12 cells induced by OS with H_2_O_2_. We believe that our L-EPS possess a number of different mechanisms of action in regards to their regulatory effect on TDH. These include the chelation of metal ions [44], the scavenging of free radicals [45], the formation of specific redox reaction (GSH) substrates [46], and specifically, the reduction of some different protein disulfate bonds (thioredoxin) [47]. However, further research is needed to fully understand the precise mechanism and clinical applications of the regulatory effect of our L-EPS on TDH.

OS mainly causes NDs by creating a disequilibrium between oxidative and antioxidative systems and altered levels of TOS and TAS [48]. TOS and TAS levels are surrogate markers of oxidative balance. They serve as a general overview of the oxidative balance and obviate the need to measure oxidant and antioxidant molecules independently [49]. Thus, we used commercial kits to gauge how TOS and TAS levels were affected by L-EPSs in PC-12 cells with H_2_O_2_-induced OS (in vitro ND model). In our study, the H_2_O_2_ group showed a significantly higher TOS level and a significantly lower TAS level than did the controls (*p* < 0.05). The L-EPSs groups obtained a significant reduction in TOS levels in the H_2_O_2_ (*p* < 0.05). The L-EPS groups caused a significant increment in TAS levels compared with those in the H_2_O_2_ group (*p* < 0.05). Arikanoglu et al. [50] and Copoglu et al. [51] examined serum and plasma TOS and TAS levels in ND patients and controls. The TOS levels were significantly higher, and the TAS levels significantly lower in ND patients than in the controls. The high TOS and low TAS levels in the H_2_O_2_ group in our study are similar to those reported by Arikanoglu et al. [50] and Copoglu et al. [51] in patients with NDs. However, while the study of Arikanoglu et al. [50] and Copoglu et al. [51] included patients with NDs, our study included PC-12 cells induced by OS with H_2_O_2_ (in vitro ND model). In a study reported by Şirin and Aslım [52], L-EPSs originating from lactic acid bacteria effectively mitigated Aβ_1–42_-induced OS in SH-SY5Y cells by preserving a balance between TOS and TAS. While the results obtained from our study are similar to those noted in that study, they differ in terms of the cell line and stimulating agent used in the research. There is one study on this subject found in the literature. This study also belongs to our group. The studies in the literature focus on the effects of probiotics on TOS and TAS levels, but these studies are not related to NDs [53,54]. Based on our findings, there is a regulatory effect of L-EPSs on TOS and TAS levels in PC-12 cells induced by OS with H_2_O_2_. It has been stated in the literature that L-EPS provides balance between TOS and TAS levels, thanks to mechanisms of action such as free radical scavenging [55], metal chelation [56], lipid peroxidation [57], electron or hydrogen donation (to ROS) [58], antioxidant stimulant, and enzyme detoxification activities [59]. This view was supported by our findings in a previous study, which implied that our L-EPSs exhibited antioxidant activities (hydroxyl and superoxide anion scavenging activity, metal ion chelating activity, and lipid peroxidation activity) [35]. In addition, the regulatory effects of our L-EPSs on TDH also contribute to the maintenance of the TOS/TAS balance.

There are two classes of the antioxidant system: the enzyme antioxidant system and the non-enzyme antioxidant system. The enzyme antioxidant system is the first-line anti-OS defense system and mainly includes SOD, CAT, GPx, and various other endogenous antioxidant enzymes (PON1) [60]. SOD is primarily responsible for the neutralization of the superoxide radical by dismutating it into H_2_O_2_ and oxygen. Peroxynitrite forms when nitric oxide and a superoxide radical enter a chemical reaction, and it shows oxidizing and nitrating properties; SOD is also responsible for detoxification [61]. CAT, a redox enzyme containing heme, disintegrates H_2_O_2_ into molecular oxygen and water in a radical-free reaction, thus providing protection against its harmful effects [62]. GPx is a selenoenzyme that reduces organic hydroperoxides (-ROOH) into alcohol and water by using reduced glutathione (GSH) as a cosubstrate [63]. PON1 disintegrates lipid peroxides and prevents plasma lipoproteins from undergoing oxidative alteration [64]. It has been reported that the levels of these enzyme antioxidant systems are reduced in patients with NDs [65,66]. We therefore investigated how L-EPSs affected PON1, SOD, CAT, and GPx antioxidant enzyme levels in PC-12 cells with H_2_O_2_-induced OS (in vitro ND model) using commercial kits. In our study, PON1, SOD, CAT, and GPx antioxidant enzyme levels were found to be significantly lower in the H_2_O_2_ group compared with the control group (*p* < 0.05). PON1, SOD, CAT, and GPx enzyme levels were considerably increased by the L-EPS groups as compared with the H_2_O_2_ group (*p* < 0.05). Romani et al. [67] and Vural et al. [68] reported that the PON1, SOD, CAT, and GPx levels were significantly lower in ND patients than in the controls. The low PON1, SOD, CAT, and GPx enzyme levels in the H_2_O_2_ group in our study are similar to those reported in patients with NDs by Romani et al. [67] and Vural et al. [68]. However, while the study of Romani et al. [67] and Vural et al. [68] included patients with NDs, our study included PC-12 cells induced by OS with H_2_O_2_ (in vitro ND model). Şirin and Aslim [52] reported that L-EPS protect SH-SY5Y cells against Aβ-mediated neurotoxicity by regulating SOD, CAT, and GPX antioxidant enzyme activities. Şengül et al. [69] used an experimental rat colitis model to investigate whether EPS-producing probiotic bacteria inhibited intestinal oxidative damage associated with the pathogenesis of NDs. All antioxidant enzyme activities (SOD, CAT, and GPx) were higher in both EPS-producing probiotic bacteria-treated groups compared with those of the colitis model group. While the results obtained from our study are similar to those obtained in these studies, they differ in terms of the cell line/model and stimulating agent used in the studies. Based on our findings, there is an inductive effect of L-EPSs on PON1, SOD, CAT, and GPx antioxidant enzyme levels in PC-12 cells induced by OS with H_2_O_2_. In the literature, the potential mechanisms by which EPSs regulate antioxidant enzyme levels are as follows: modulation of antioxidant enzyme expression via NRF2-KEAP1 [70], preservation of antioxidant enzyme activity [71], enhancement of endogenous antioxidants [72], and scavenging of intracellular ROS [73]. To determine the potential mechanism of the inducing effect of our L-EPS on PON1, SOD, CAT and GPx antioxidant enzyme levels, additional experiments were conducted to determine the scavenging of intracellular ROS and the modulation of antioxidant enzyme expression via NRF2-KEAP1.

The initial and developmental stages of NDs mainly include the ROS, namely superoxide anion radical, H_2_O_2_, the hydroxyl radical, and nitric oxide. Nitric oxide has a secondary signal messenger function; it enters a reaction with oxygen, forming a peroxynitrite radical, a compound that directly alters and damages the aromatic rings of aminoacid residues. Peroxynitrite reacts with sulfhydryls, lipids, proteins, and DNA [74]. Studies show that ROS might play a pivotal role, since elevated levels of OS are frequently detected in the brains of individuals with NDs [75,76]. We therefore investigated how L-EPSs affected intracellular ROS levels in PC-12 cells with H_2_O_2_-induced OS (in vitro ND model) using flow cytometry. In our study, the intracellular ROS level was found to be significantly higher in the H_2_O_2_ group compared with the control group (*p* < 0.05). The intracellular ROS levels were substantially reduced upon treatment with L-EPS (*p* < 0.05). Zhang et al. 61] showed that AD patients have significantly elevated levels of ROS in the brain, experiencing OS. The high intracellular ROS levels in the H_2_O_2_ group found in our study are similar to those reported by Zhang et al. [77] in patients with AD. However, while the study of Zhang et al. [77] included patients with AD, our study included PC-12 cells induced by OS with H_2_O_2_ (in vitro ND model). In research conducted by Zhang et al. [78], the EPS from *L. plantarum* YW11 displayed a remarkable scavenging ability, with IC_50_ values against hydroxyl radical of 75.10% and 1.22 mg/mL, and against superoxide anion radical of 62.71% and 1.54 mg/mL. In a separate study by Adesulu-Dahunsi et al. [79], two EPS types from *L. plantarum* demonstrated a moderate hydroxyl radical scavenging capability. Specifically, as the concentration of EPS-OF101 increased from 0.5 mg/mL to 4 mg/mL, its scavenging ability rose from 23% to 45.3%. Similarly, for EPS-YO175, the scavenging activity grew from 43.2% to 89.4% within the same concentration range. While the results obtained from our study are similar to those reported in these studies, they differ in terms of the bacterial strains from which EPS is used, as well as in the concentration of EPSs. Based on our findings, there is an inhibitory effect of L-EPSs on the intracellular ROS level in PC-12 cells induced by OS with H_2_O_2_. It has been stated in the literature that L-EPSs are effective in reducing intracellular ROS levels through the mechanisms of direct ROS scavenging [80], metal ion chelation [81], antioxidant enzyme activation [82], stabilization of the cell membrane [83], modulation of signaling pathways [84], reducing lipid peroxidation [85], and increasing antioxidant capacity [86]. In addition, this study revealed that one of the potential mechanisms of the inducing effect of our L-EPS on PON1, SOD, CAT, and GPx antioxidant enzyme levels is the scavenging of intracellular ROS. To determine the potential mechanism of the inhibitory effect of our L-EPS on intracellular ROS, additional experiment were conducted to determine the modulation of antioxidant enzyme expression via NRF2-KEAP1.

The NRF2-ARE pathway is an important biochemical step in the pathogenesis of NDs. The ARE is a consensus sequence RTGACnnnGC enhancer element found in the 5′ flanking region of multiple phase II detoxifying and antioxidant genes. The actin-binding protein KEAP1 is connected to NRF2, which is found in the cytoplasm. Before NRF2 is disintegrated by proteasomes, it is polyubiquitinated by the KEAP1, which is a Cul3-based E3 ligase. Once exposed to OS or electrophilic agents reactive with KEAP1, NRF2 is stabilized, increasing the levels of NRF2 protein and leading to NRF2 accumulation in the nucleus. In the nucleus, NRF2 forms dimers with small MAF proteins and binds ARE, a promoter of the expression of several genes participating in detoxification and antioxidant processes (GCLC, HO-1, and NQO1) [87]. Recent studies highlight the significance of the NRF2-KEAP1 pathway as a potential therapeutic target for NDs [88]. Therefore, we employed qRT-PCR and ELISA to study the effect of L-EPSs on GCLC, HO-1, NRF2, NQO1, and KEAP1 gene and protein expression levels in PC-12 cells with H_2_O_2-_induced OS (in vitro ND model). In our study, the H_2_O_2_ group exhibited significantly lower GCLC, HO-1, NRF2, and NQO1 gene and protein expression levels than did the controls (*p* < 0.05). The H_2_O_2_ group showed a significantly increased KEAP1 gene and protein expression level than did the control (*p* < 0.05). The reduction of GCLC, HO-1, NRF2, NQO1 gene and protein expression levels was significantly increased by the L-EPS groups (*p* < 0.05). The increase in the KEAP1 gene and the protein expression level was significantly decreased by the L-EPSs groups (*p* < 0.05). Kanninen et al. [89] observed that the amounts of Aβ dramatically increased, while the levels of NRF2 significantly decreased as the mice aged. This was accompanied by the decreased expression of three known targets of the NRF2 pathway, NQO1, GCLC, and GCLM. Similary, an age-dependent decrease in the mRNA levels of NRF2 was shown in the cortex of wild-type mice [90]. The high KEAP1 and low GCLC, HO-1, NRF2, and NQO1 gene and protein expression levels in the H_2_O_2_ group in our study are similar to those reported by Kanninen et al. [89] and Gureev et al. [90] in aged mice. However, while the study of Kanninen et al. [89] and Gureev et al. [90] included aged mice, our study included PC-12 cells induced by OS with H_2_O_2_ (in vitro ND model). Moreover, they differ in terms of the stimulating agent used in the studies. According to a study by Wang et al. [11], EPSs (EPS103) from *L. plantarum* JLAU103 activated the antioxidation enzymes and the NRF2/KEAP1 pathway, thereby exhibiting a protective effect against OS mediated by cyclophosphamide in immune-suppressed mice. In a study by Zhang et al. [10], *B. amyloliquefaciens* lysate (BAL1) with EPS content favored the nuclear transfer of NRF2 in human embryonic fibroblasts (CCC-ESF-1), activating downstream antioxidant enzyme gene transcription and boosting the antioxidant effectiveness of antioxidant enzymes to eliminate higher amounts of intracellular ROS. While the results obtained from our study are similar to those found in these studies, they differ in terms of the bacterial strains from which EPS is used, the stimulating agent, and the cell line/model. Based on our findings, there is a regulatory effect of L-EPSs on GCLC, HO-1, NRF2, NQO1, and KEAP1 protein expression levels in PC-12 cells induced by OS with H_2_O_2_. In addition, this study revealed that one of the potential mechanisms of the modulatory effect of our L-EPS on PON1, SOD, CAT, and GPx antioxidant enzyme levels and intracellular ROS is the modulation of antioxidant enzyme expression via NRF2-KEAP1.

Our research provided unequivocal evidence for the activation of the NRF2 pathway triggered by L-EPSs. The interaction of L-EPSs with toll-like (TLR) receptors promotes the release of signaling molecules that lead to the activation of the Nrf2-Keap signaling pathway [91]. This activation is mechanistically underpinned by the L-EPSs-mediated downregulation of the KEAP1 protein, an intrinsic inhibitor of NRF2. By attenuating KEAP1 expression, L-EPSs effectively obviate the NRF2-KEAP1 binding, thereby liberating NRF2 to translocate to the nucleus. Subsequent to this molecular event, there is a pronounced augmentation in the transcriptional activity of antioxidant response element (ARE)-driven genes, specifically those encoding antioxidant enzymes such as GCLC, HO-1, and NQO1. This enhanced enzymatic profile confers resistance against OS instigated by H_2_O_2_. Furthermore, our findings elucidate that L-EPSs also potentiate the expression of other antioxidant enzymes, namely PON1, SOD, CAT, and GPx. This upregulation, we posit, is intricately linked to the modulatory effects exerted on the NRF2-KEAP1 axis by the L-EPSs. This suggests that the L-EPSs not only bolster the primary NRF2-mediated defense, but also augment secondary antioxidant systems, underscoring their comprehensive protective role against OS. 

The potential limitations of this research can be highlighted as follows: 1. The specific cell line used: this study utilized PC12 cells, which are derived from a pheochromocytoma of the rat adrenal medulla. While PC12 cells are a common model for neuronal studies, results obtained from these cells might not entirely replicate those from primary neuronal cultures or in vivo models. The cellular processes and responses in PC12 might differ from those of other types of neuronal or non-neuronal cells. 2. In vitro vs. in vivo experiments: conducting experiments in cell cultures (in vitro) does not always translate to the same results in live organisms (in vivo). This is due to the complexity of biological systems, the presence of multiple interacting cell types, and the myriad of other factors present in an organism that could influence outcomes. 3. The potential confounding variables: As in any in vitro research, variables such as the concentration of L-EPSs used, the duration of treatment, and other experimental conditions could influence the results. It is also possible that the observed effects are not solely attributable to the L-EPSs, but might result from other, unmeasured variables. 4. The limitations regarding mechanistic understanding: while the NRF2-KEAP1 pathway’s involvement is evident from the study, there may be other cellular pathways influenced by L-EPSs that were not explored in this research. 5. Biases in study design: the potential for biases exists in any study. For example, if the study did not include appropriate controls, or if the data were interpreted without considering all potential explanations, it could introduce bias. The method of L-EPSs extraction, purification, and verification could also introduce variability. 6. Generalizability: since the study focuses on lactic acid bacteria-derived L-EPSs, the findings might not be applicable to L-EPSs derived from other microbial sources. 7. Dosage and response variability: the effect of varying concentrations of L-EPSs on PC12 cells might not have been comprehensively evaluated. This is essential to understand the dose–response relationship and potential therapeutic windows.

## 5. Conclusions

Our study sheds light on the crucial role that L-EPSs play in regulating H_2_O_2_-induced OS, a known factor in the pathogenesis of many NDs. Notably, H_2_O_2_-induced OS is intricately managed by these L-EPSs in a multi-faceted manner. First, they work towards restoring and maintaining the balance of TDH and the TOS/TAS. The importance of this equilibrium cannot be overstated, as any imbalance can lead to cellular dysfunction and eventual degeneration. Furthermore, our findings highlight the capacity of L-EPSs to promote the activities of several antioxidant enzymes, including, but not limited to, PON-1, SOD, CAT, and GPx. These enzymes are fundamental in neutralizing harmful oxidative radicals and reducing intracellular ROS levels. A decline in ROS levels signifies a decrease in potential cellular damage and a step towards cellular stability. Moreover, our study delves into the molecular mechanisms by which L-EPSs exert their protective effects. We found that L-EPSs play a pivotal role in modulating key genes and proteins within the OS-related NRF2-KEAP1 pathway. Specifically, the upregulation of protective genes like GCLC, HO-1, NRF2, and NQO1, coupled with the modulation of KEAP1, underscores the holistic approach L-EPSs take in managing OS. In essence, this comprehensive examination emphasizes the potential of L-EPSs as a therapeutic agent in mitigating OS, particularly H_2_O_2_-induced stress. This could pave the way for novel therapeutic strategies targeting the early stages of NDs. Further studies are warranted to corroborate these findings in diverse cell models and in vivo systems.

## Figures and Tables

**Table 1:** Primers used in qRT-PCR.

Gene Name	Forward Primer 5′→3′	Reverse Primer 5′→3′
GCLC	GTGGACACCCGATGCAGTAT	TCATCCACCTGGCAACAGTC
HO-1	GCTCTATCGTGCTCGCATGA	AATTCCCACTGCCACGGTC
KEAP	TGGGCGTGGCAGTGCTCAAC	GCCCATCGTAGCCTCCTGCG
NRF2	GCTGCCATTAGTCAGTCGCTCTC	ACCGTGCCTTCAGTGTGCTTC
NQO1	ACATCACAGGGGAGCCGAAGGACT	GGCACCCCAAACCAATACAATG
GAPDH	CAACTCCCTCAAGATTGTCAGCAA	GGCATGGACTGTGGTCATGA

**Table 2:** The expression levels of the proteins GCLC, HO-1, NRF2, NQO1, and KEAP1.

Treatment	Relative Protein Levels (% of Control)				
	GCLC	HO-1	NRF2	NQO1	KEAP1
Control	100 ± 3	100 ± 5	100 ± 6	100 ± 3	100 ± 1
H_2_O_2_	30 ± 2 ^#^	33 ± 4 ^#^	25 ± 3 ^#^	36 ± 2 ^#^	225 ± 2 ^#^
100 μg/mL B3 L-EPS	33± 1	34± 5	36 ± 7 *	36± 1	217 ± 3
250 μg/mL B3 L-EPS	46 ±4 *	44 ±6 *	53 ± 1 *	39 ±5	208 ± 1 *
500 μg/mL B3 L-EPS	59 ± 5 *	56 ± 1 *	70 ± 2 *	51± 4 *	162 ± 5 *
1000 μg/mL B3 L-EPS	79 ± 2 *	76 ± 2 *	91 ± 4 *	69 ± 2 *	133 ± 3 *
1250 μg/mL B3 L-EPS	93 ± 6 *	89 ± 3 *	106 ± 6 *	82± 1 *	98 ± 4 *
100 μg/mL GD2 L-EPS	38± 5	35± 1	34 ± 2 *	38± 5	220 ± 5
250 μg/mL GD2 L-EPS	48 ± 3 *	46 ±2 *	51 ± 4 *	45 ±4 *	213 ± 3 *
500 μg/mL GD2 L-EPS	65 ± 1 *	59 ± 3 *	68± 1 *	56± 1 *	167 ± 1 *
1000 μg/mL GD2 L-EPS	86 ± 7 *	79 ± 6 *	87 ± 3 *	75 ± 3 *	137 ± 2 *
1250 μg/mL GD2 L-EPS	98 ± 2 *	93 ± 8 *	102 ± 4 *	89 ± 4 *	106 ± 3 *

Values are expressed as mean ± SD. Tukey’s test was applied, if *p* < 0.05. ^#^ Significant difference from the control (*n*:3 for each bar). * Significant difference from the H_2_O_2_ (*n*:3 for each bar).

## Data Availability

The data presented in this study are available on request from the corresponding author.
